# Supplementary material for: Influences of familiarity and recollection on value-based decision-making
Source: PLoS One. 2025 May 14;20(5):e0322632. doi: 10.1371/journal.pone.0322632 (PMC12077727; doi:10.1371/journal.pone.0322632)
Supplement: S1 Appendix — (DOCX) [file pone.0322632.s001.docx]

# Influences of familiarity and recollection on value-based decision-making

Supporting information

**Experiment 2 – Online Sample**

The behavior from the online sample indicated a failure of a large proportion of these participants to comply with the basic task instructions and that they did not engage with the memory-related aspects of the task. As a consequence, these data are challenging to interpret and bear limited relevance to the hypothesis we intended to test. However, in an effort to provide full reporting and to limit publication bias (i.e. the ‘file drawer problem’), we are reporting the planned analyses that we conducted on these data. We believe these findings may be informative, particularly for those wishing to reproduce our results or design similar experiments. We discuss these implications following our report of the results.

***Experiment 2 – Memory and Decision-Making Performance***

As with the original experiment, we first examined the effect of our repetition manipulation on memory measures (Supplementary Table 1). Familiarity, source confidence and accuracy increased with the number of presentations. However, the RPR did not change, and the mean RPR did not differ from what would be expected according to chance for any presentation level (*P’*s ≥ 0.7).

**Table S1.** Familiarity and source memory measures across presentations

| **Measure** | **Lure (M, SD)** | **One presentation (M, SD)** | **Three presentations (M, SD)** | **Statistics** |
| --- | --- | --- | --- | --- |
| Familiarity ratings | 2.78 (0.60)* | 3.21 (0.46) * | 3.67 (0.53) * | *F* (2,174) =112.90, *P <* 0.0001, η^2^= 0.56 |
| Source Confidence | 2.72 (0.99)* | 2.84 (0.95)* | 2.94 (0.88)* | *F* (2,174) = 35.65, *P <* 0.0001, η^2^= 0.29 |
| Retrieved probability of reward (RPR) | 0.50 (0.08) | 0.50 (0.08) | 0.50 (0.08) | *F* (2,174) = 0.69, *P* = 0.5, η^2^= 0.01 |
| Source Accuracy | - | 0.26 (0.04) | 0.27 (0.05) | *t* (87) = 2.03, *P* = 0.04, *d* = 0.22 |

* P < 0.001, Bonferroni post-hoc repeated measures t-test against all other levels

We next evaluated the effects of familiarity, source memory and lure status, as well as their interactions, on participants betting decisions in a model that included both target and lure trials. Unlike in Experiment 1, there was no significant effect for the intercept term (OR = 0.86, 95% CI: 0.57-1.28, P = 0.4), indicating that participants did not show any overall bias towards betting or passing.

Next, we assessed how familiarity influenced decisions during the memory-based betting task. We replicated the strong overall effect for familiarity to increase betting rates (Figure S1a; OR = 1.28, 95% CI: 1.15-1.42, P < 0.0001), and the tendency for participants to bet less frequently on lure items in general (OR = 0.67, 95% CI: 0.58-0.77, P < 0.0001). However, unlike in Experiment 1, there was a significant interaction between lure status and familiarity such that the influence of familiarity was reduced for these lure items (Figure S1b; OR = 0.87, 95% CI: 0.81-0.93, P < 0.0001).

We also examined how betting rates were related to source memory for reward information across all trials. There was a significant effect of source confidence (OR = 1.07, 95% CI: 1.01-1.13, P = 0.02), but no significant effect for RPR (OR = 1.04, 95% CI: 0.98-1.11, P = 0.2). Unlike familiarity ratings, there was no significant interaction between these effects and the lure status of items (P’s ≥ 0.4).

We also tested whether these effects differed by the order of the betting and memory judgment tasks within the experiment (i.e. ‘Memory-Betting’ or ‘Betting-Memory’). There was no change in the overall rate of betting based on task order (OR = 0.86, 95% CI: 0.49-1.49, P = 0.6) Nor were there any changes in the the effects of familiarity, RPR or source confidence as a function of task order (P’s ≥ 0.1). However, participants were much more likely to bet on lure items if they had completed the memory judgment task before betting (OR = 1.46, 95% CI: 1.21-1.77, P = 0.0001), indicating that either exposure or probing participants about their memory for these lure items changed their willingness to engage with a bet.

Next, we examined how retrieval of source memories impacted betting on targets by examining the main effect of source accuracy and its interactions with source confidence and RPR (Figure S1c). There was no main effect of source accuracy on betting rates, nor any significant two- or three-way interactions between source accuracy, RPR and source confidence (*P*’s ≥ 0.09). Thus, there was no clear evidence that participants were using information retrieved from source memory in this betting task.

We also examined the interactions of source memory judgments and familiarity, as part of our planned analyses. Betting rates tended to increase more for items with a higher RPR and higher familiarity – but this trend was not statistically significantly (Figure S1d; OR = 1.04, 95% CI: 1.00-1.07, P = 0.06). There were also no significant interactions between familiarity and source confidence, or any significant three-way interaction between familiarity, source confidence and RPR (P’s ≥ 0.5). The two-way interaction of source confidence and RPR was also not significant (OR = 1.01, 95% CI: 0.97-1.05, P = 0.7). Thus, not surprisingly, given the lack of evidence for a use of source memory, participants’ choices were not clearly influence by source confidence and the subjective probability of reward and there was no evidence interaction with familiarity.

We finally examined the role of familiarity and source memory on betting for lure items (Figure S1e). Unlike in Experiment 1, there was no significant effect for familiarity in lure items alone (OR = 1.08, 95% CI: 0.97-1.21, P = 0.2). There were also no main effects of source confidence or RPR (P’s ≥ 0.5), nor any significant two- or three-way interactions between these source memory signals with each other or with familiarity (P’s > 0.2). There was thus little indication of what, if anything, changed participants’ betting behavior for lure items in the online sample in Experiment 2.

**Figure S1.** Behavior from online sample in Experiment 2. Panels **a.** and **b.** show betting rates as a function of familiarity for targets (**a**) and lures(**b**). Green and blue lines show individual participants, mean and standard error shown in dashed black lines. **c.** Betting rates as a function of the three-way interaction of source accuracy (correct or error), confidence (low: source confidence rating ≤ 3, high: source confidence rating > 3) and retrieved probability of reward (RPR) (10% or 40% (10-40), 60 or 90% (60-90)). Panels **d.** and **e.** both show interaction between familiarity ratings (low: rating ≤ 3, high: rating > 3), and source confidence for target items (**d**), and for lures (**e**).

***Experiment 2 – Comparison of memory performance across samples***

The betting behavior in the online and in-laboratory samples for Experiment 2 differed considerably. While both samples showed the expected increase in betting on subjectively familiar items, the influence of source information on betting rates was generally much clearer in the in-laboratory sample. We suspected that this difference might reflect a disparity in the extent to which participants in these two samples engaged in the memory judgments in this task. To answer this question, we compared these two samples on source memory accuracy and recognition performance.

Participants in the in-laboratory sample performed significantly better on memory tasks compared to the online sample. We estimated the accuracy of participants’ source memory by calculating frequency in which they accurately identified the correct source during the source judgment phase of the task (Figure S2a). The source accuracy of both samples was significantly greater than chance in both the online sample (one-sample, one-tailed t-test: *t*(87) = 3.91, P < 0.0001, Cohen’s *d* = 0.42), and in-laboratory sample (*t*(35) = 6.42, P < 0.0001, Cohen’s *d* = 1.07)*.* However, overall, the source accuracy of the in-laboratory sample was also significantly greater than their online counterparts (independent samples, two-tailed t-test: *t*(122) = 5.19, P < 0.0001, Cohen’s *d* = 1.03). We also examined performance on the recognition memory test, calculated as a d-prime score (Figure S2b). The d-prime score was significantly greater than zero in the online sample (one sample, one-tailed t-test: *t*(87) = 9.84, P < 0.0001, Cohen’s *d* = 1.05), and in-laboratory samples (*t*(122) = 14.61, P < 0.0001, Cohen’s *d* = 1.56). However, as with source memory performance, the in-laboratory d-prime scores were significantly greater than the online sample (independent samples, two-tailed t-test: *t*(122) = 6.26, P < 0.0001, Cohen’s *d* = 1.24).

**Figure S2.** Comparison of performance in recognition memory and source memory between Experiment 2 samples. **a.** Source accuracy for target items, chance rate (25%) is indicated by the red dashed line. **b.** d-prime based on hit and false alarm rates in recognition memory judgements. *** *P* < 0.0001, two-tailed between-subjects t-test, ### *P* < 0.0001, two-tailed one sample t-test against chance.

***Experiment 2 – relationship of familiarity and source confidence***

We carried out the same test of the relationship between source memory and familiarity in the online sample which we had also tested for the in-laboratory participants. As with that sample, neither a linear nor exponential function was overwhelmingly a better fit to these data based on the total negative log-likelihood across participants (online: linear = 2464.89, exponential = 2464.97. Similarly, the linear model was a better fit for only a slightly larger percentage of participants (online: 55.7%).

We also compared the slopes for the linear function fit to the familiarity and source confidence ratings for target items where participants made correct and incorrect source judgments, and for lure items. Similar to the in-laboratory sample, the slope of this function was higher on both correct and erroneous source judgments compared to lures (Figure S3; within-subject, two-tailed t-test: t(87) ≥ 4.92, *P*’s ≤ 0.0001, Cohen’s *d* ≥ 0.52, corrected). The slope of this function was also numerically higher for correct source judgments compared to errors, though this effect did not survive correction for multiple comparisons (t(87) = 2.13, *P* = 0.1, Cohen’s *d* = 0.23, corrected).

**Figure S3.** Slopes for a linear function fit to the relationship between familiarity and source confidence for different item types (lures, targets with correct and incorrect source judgments). *** *P* < 0.0001, two-tailed within-subjects t-test, corrected for multiple comparisons.

***Experiment 2 – Online Sample Discussion***

The behavior of this online sample indicated lack of compliance with task instructions. In particular, these participants did not significantly modulate their betting rates according to the RPR. In a value-based decision-making paradigm such as this, there is no one ‘right’ way to complete the task, as the problem is inherently one of personal choice and subjective assessment. However, we would expect that decisions should be modulated by the RPR– if participants were trying to recall the source of each item during the memory-based decision-making phase. These online participants also performed significantly worse in source memory and familiarity judgments compared to the in-laboratory sample. Thus, the on-line participants seemed to be less engaged with the memory-related aspects of the task and/or less willing to use information stored in memory to make choices. As our hypotheses assumed participants engagement with the task and active use of memory during decision-making, we believe these data have limited value for answering our scientific questions. However, these results do provide some useful insight into how small differences in task design and administration can substantially alter results, which we explore below.

There are many potential reasons for these differences from the in-laboratory sample, who completed the same experiment, and the participants in Experiment 1 who completed a similar task online. The task itself was longer than in Experiment 1, and the memory judgments were more repetitive and had a greater separation from the decision task. Our in-laboratory participants were also monitored by an experimenter throughout the task, while online participants completed the experiment without any external supervision. Online participants are also likely more motivated to complete experiments quickly and devote less time and cognitive effort to demanding recollection tasks so that they may complete other tasks and earn more money in a shorter amount of time. The participants in the online sample were also likely more experienced with these kinds of tasks, which may have made them less interested in this particular experiment.

Notably, there were some commonalities in the results for the online and in-laboratory samples. There was a small, albeit noisy, bias towards betting on more subjectively familiar target stimuli in the online sample. The steepness for the relationship between source confidence and familiarity also increased for targets compared to lures, as in the in-laboratory sample. Thus, effects of familiarity might be more easily detectable than those that depend on a more demanding and complicated retrieval process.

The differences between the online and in-laboratory sample indicate important considerations for designing studies examining interactions between memory and decision-making. Future work will need to ensure that participants are equally motivated to engage in the value-based decision-making aspects of the task as well as memory judgments that help support interpretation of decision behavior. These data also speak to how small changes in the way an experiment is conducted can dramatically change the results. Experimenters must exercise caution when interpreting replication failures and consider how seemingly small changes in the sample or task administration can have cascading consequences for compliance with instructions and task strategy.

***Experiments 1 and 2 – individual differences***

In experiment 1 we found negative relationships between recognition memory performance (measured as d-prime scores) and mean overall betting rates (Pearson’s correlation: r(42) = -0.39, P = 0.009), as well as between source accuracy and betting rates (r(42) = -0.50, P = 0.0005). However, we did not find the same relationship for recognition memory in the in-laboratory sample for experiment 2: (d-prime and betting rates: r(34) = 0.15, P = 0.4), and this effect was trending for source accuracy and betting rates (r(34) = -0.29, P = 0.09). The online sample in experiment 2 showed only a trending relationship between recognition memory and betting rates in the same direction as in experiment 1 (d-prime and betting rates: r(86) = -0.19, P = 0.08), and a weaker but significant relationship between source accuracy and betting rates: r(86) = -0.31, P = 0.003).

**Figure S4**. Scatterplots showing relationship between mean betting rate and performance of familiarity task (d-prime) and source accuracy across two experiments with online and in-laboratory samples. Statistics represent Pearson correlation coefficients.

Table S2. LME 1.1 – All Trials

| Term | OR | 95% CI LL | 95% CI UL | P-value |
| --- | --- | --- | --- | --- |
| (Intercept) | 0.38 | 0.20 | 0.71 | 0.003 |
| lure_1 | 0.78 | 0.66 | 0.92 | 0.003 |
| familiarity | 2.93 | 2.04 | 4.19 | <0.0001 |
| familiarity*lure_1 | 0.81 | 0.57 | 1.13 | 0.2 |

Table S3. LME 1.2 – Lures only

| Term | OR | 95% CI LL | 95% CI UL | P-value |
| --- | --- | --- | --- | --- |
| (Intercept) | 0.22 | 0.10 | 0.46 | <0.0001 |
| familiarity | 1.50 | 1.08 | 2.08 | 0.01 |

Table S4. LME 1.3.- High Familiarity target items

| Term | OR | 95% CI LL | 95% CI UL | P-value |
| --- | --- | --- | --- | --- |
| (Intercept) | 1.99 | 0.90 | 4.37 | 0.09 |
| RPR | 2.13 | 1.25 | 3.64 | 0.005 |
| familiarity | 1.79 | 1.38 | 2.32 | <0.0001 |
| sourceConf | 1.52 | 1.24 | 1.85 | <0.0001 |
| sourceAcc_1 | 0.99 | 0.77 | 1.28 | 0.9 |
| RPR*familiarity | 1.08 | 0.93 | 1.24 | 0.3 |
| RPR*sourceConf | 1.20 | 1.00 | 1.44 | 0.05 |
| familiarity*sourceConf | 1.11 | 1.00 | 1.24 | 0.06 |
| RPR:sourceAcc_1 | 1.07 | 0.80 | 1.41 | 0.6 |
| sourceConf*sourceAcc_1 | 0.99 | 0.78 | 1.26 | 0.9 |
| RPR*familiarity*sourceConf | 0.96 | 0.87 | 1.06 | 0.4 |
| RPR*sourceConf*sourceAcc_1 | 1.22 | 0.93 | 1.59 | 0.1 |

Table S5. LME 2.1 – In-laboratory Cohort – All trials

| Term | OR | 95% CI LL | 95% CI UL | P-value |
| --- | --- | --- | --- | --- |
| (Intercept) | 0.38 | 0.24 | 0.60 | <0.0001 |
| lure_1 | 0.39 | 0.27 | 0.54 | <0.0001 |
| RPR | 1.36 | 1.17 | 1.58 | 0.0001 |
| familiarity | 2.07 | 1.66 | 2.59 | <0.0001 |
| sourceConf | 1.38 | 1.20 | 1.59 | <0.0001 |
| task_RateBet | 1.83 | 0.97 | 3.43 | 0.06 |
| lure_1*RPR | 0.86 | 0.76 | 0.99 | 0.03 |
| lure_1*familiarity | 0.82 | 0.69 | 0.96 | 0.01 |
| lure_1*sourceConf | 0.91 | 0.78 | 1.06 | 0.2 |
| lure_1*task_RateBet | 1.94 | 1.27 | 2.97 | 0.002 |
| RPR*task_RateBet | 1.03 | 0.87 | 1.23 | 0.7 |
| familiarity*task_RateBet | 0.71 | 0.54 | 0.93 | 0.01 |
| sourceConf*task_RateBet | 0.98 | 0.81 | 1.19 | 0.8 |

Table S6. LME 2.2 – In-laboratory Cohort – Target items

| Term | OR | 95% CI LL | 95% CI UL | P-value |
| --- | --- | --- | --- | --- |
| (Intercept) | 0.46 | 0.31 | 0.70 | 0.0002 |
| RPR | 1.18 | 1.02 | 1.36 | 0.02 |
| familiarity | 1.80 | 1.44 | 2.24 | <0.0001 |
| sourceConf | 1.43 | 1.22 | 1.68 | <0.0001 |
| sourceAcc_1 | 1.08 | 0.89 | 1.30 | 0.4 |
| task_RateBet | 1.99 | 1.17 | 3.38 | 0.01 |
| RPR*familiarity | 1.11 | 1.02 | 1.20 | 0.01 |
| RPR*sourceConf | 1.11 | 1.02 | 1.22 | 0.02 |
| Familiarity*sourceConf | 1.03 | 0.94 | 1.13 | 0.5 |
| RPR*sourceAcc_1 | 1.18 | 1.04 | 1.34 | 0.01 |
| sourceConf*sourceAcc_1 | 0.95 | 0.82 | 1.09 | 0.5 |
| RPR*task_RateBet | 1.10 | 0.92 | 1.30 | 0.3 |
| Familiarity*task_RateBet | 0.85 | 0.63 | 1.13 | 0.2 |
| sourceConf*task_RateBet | 1.00 | 0.81 | 1.23 | 0.9 |
| sourceAcc_1*task_RateBet | 0.98 | 0.76 | 1.27 | 0.9 |
| RPR*familiarity*sourceConf | 1.11 | 1.02 | 1.20 | 0.01 |
| RPR*sourceConf*sourceAcc_1 | 1.26 | 1.10 | 1.45 | 0.001 |

Table S7. LME 2.3 – In-laboratory Cohort – Lure items

| Term | OR | 95% CI LL | 95% CI UL | P-value |
| --- | --- | --- | --- | --- |
| (Intercept) | 0.11 | 0.04 | 0.26 | <0.0001 |
| RPR | 1.23 | 1.03 | 1.47 | 0.02 |
| familiarity | 1.64 | 1.35 | 1.99 | <0.0001 |
| sourceConf | 0.98 | 0.81 | 1.19 | 0.8 |
| task_RateBet | 3.32 | 1.19 | 9.28 | 0.02 |
| RPR*familiarity | 0.91 | 0.81 | 1.01 | 0.08 |
| RPR*sourceConf | 1.13 | 1.02 | 1.26 | 0.02 |
| Familiarity*sourceConf | 0.94 | 0.85 | 1.04 | 0.2 |
| RPR*task_RateBet | 0.98 | 0.78 | 1.23 | 0.8 |
| Familiarity*task_RateBet | 0.71 | 0.56 | 0.90 | 0.005 |
| sourceConf*task_RateBet | 1.25 | 0.94 | 1.64 | 0.1 |
| RPR*familiarity:sourceConf | 1.01 | 0.93 | 1.11 | 0.8 |

Table S8. LME 2.4 – In-laboratory Cohort – Lure items, Betting-Rating Order

| Term | OR | 95% CI LL | 95% CI UL | P-value |
| --- | --- | --- | --- | --- |
| (Intercept) | 0.10 | 0.04 | 0.29 | <0.0001 |
| RPR | 1.29 | 1.08 | 1.55 | 0.005 |
| familiarity | 1.67 | 1.35 | 2.07 | <0.0001 |
| sourceConf | 1.01 | 0.81 | 1.26 | 0.9 |
| RPR*familiarity | 0.80 | 0.64 | 1.00 | 0.05 |
| RPR*sourceConf | 1.07 | 0.88 | 1.29 | 0.5 |
| Familiarity*sourceConf | 0.97 | 0.84 | 1.12 | 0.7 |
| RPR*familiarity:sourceConf | 1.05 | 0.92 | 1.19 | 0.5 |

Table S9. LME 2.4 – In-laboratory Cohort – Lure items, Rating-Betting Order

| Term | OR | 95% CI LL | 95% CI UL | P-value |
| --- | --- | --- | --- | --- |
| (Intercept) | 0.34 | 0.19 | 0.63 | 0.0005 |
| RPR | 1.20 | 1.05 | 1.38 | 0.007 |
| familiarity | 1.17 | 0.99 | 1.39 | 0.06 |
| sourceConf | 1.24 | 1.02 | 1.51 | 0.03 |
| RPR*familiarity | 0.93 | 0.81 | 1.06 | 0.3 |
| RPR*sourceConf | 1.15 | 1.01 | 1.30 | 0.04 |
| Familiarity*sourceConf | 0.92 | 0.80 | 1.06 | 0.2 |
| RPR*familiarity:sourceConf | 1.02 | 0.90 | 1.15 | 0.7 |

Table S10. LME 2.1 – Online Cohort – All trials

| Term | OR | 95% CI LL | 95% CI UL | P-value |
| --- | --- | --- | --- | --- |
| (Intercept) | 0.86 | 0.57 | 1.28 | 0.4 |
| lure_1 | 0.67 | 0.58 | 0.77 | <0.0001 |
| RPR | 1.04 | 0.98 | 1.11 | 0.2 |
| familiarity | 1.28 | 1.15 | 1.42 | <0.0001 |
| sourceConf | 1.07 | 1.01 | 1.13 | 0.02 |
| order_RateBet | 0.86 | 0.49 | 1.49 | 0.6 |
| lure_1*RPR | 1.01 | 0.95 | 1.07 | 0.7 |
| lure_1*familiarity | 0.87 | 0.81 | 0.93 | <0.0001 |
| lure_1*sourceConf | 0.98 | 0.92 | 1.04 | 0.4 |
| lure_1*task_RateBet | 1.46 | 1.21 | 1.77 | 0.0001 |
| RPR*task_RateBet | 1.06 | 0.98 | 1.15 | 0.1 |
| familiarity*task_RateBet | 0.94 | 0.82 | 1.08 | 0.4 |
| sourceConf*task_RateBet | 1.05 | 0.97 | 1.14 | 0.2 |

Table S11. LME 2.2 – Online Cohort – Target items

| Term | OR | 95% CI LL | 95% CI UL | P-value |
| --- | --- | --- | --- | --- |
| (Intercept) | 0.89 | 0.61 | 1.32 | 0.6 |
| RPR | 1.04 | 0.98 | 1.10 | 0.2 |
| familiarity | 1.22 | 1.10 | 1.36 | 0.0002 |
| sourceConf | 1.06 | 0.99 | 1.14 | 0.09 |
| sourceAcc_1 | 1.00 | 0.89 | 1.12 | 0.9 |
| task_RateBet | 0.92 | 0.54 | 1.56 | 0.8 |
| RPR*familiarity | 1.04 | 1.00 | 1.07 | 0.06 |
| RPR*sourceConf | 1.01 | 0.97 | 1.05 | 0.7 |
| Familiarity*sourceConf | 0.99 | 0.95 | 1.03 | 0.6 |
| RPR*sourceAcc_1 | 0.99 | 0.91 | 1.07 | 0.8 |
| sourceConf*sourceAcc_1 | 1.02 | 0.94 | 1.12 | 0.6 |
| RPR*task_RateBet | 1.06 | 0.97 | 1.15 | 0.2 |
| familiarity*task_RateBet | 1.01 | 0.87 | 1.17 | 0.9 |
| sourceConf*task_RateBet | 1.07 | 0.98 | 1.17 | 0.1 |
| sourceAcc_1*task_RateBet | 1.02 | 0.87 | 1.20 | 0.8 |
| RPR*familiarity:sourceConf | 1.01 | 0.97 | 1.05 | 0.5 |
| RPR*sourceConf:sourceAcc_1 | 1.00 | 0.92 | 1.08 | 0.9 |

Table S12. LME 2.3 – Online Cohort – Lure items

| Term | OR | 95% CI LL | 95% CI UL | P-value |
| --- | --- | --- | --- | --- |
| (Intercept) | 0.52 | 0.36 | 0.75 | 0.0005 |
| RPR | 1.03 | 0.96 | 1.10 | 0.5 |
| Familiarity | 1.08 | 0.97 | 1.21 | 0.1 |
| sourceConf | 1.01 | 0.94 | 1.09 | 0.8 |
| task_RateBet | 1.25 | 0.67 | 2.34 | 0.5 |
| RPR*familiarity | 1.01 | 0.96 | 1.07 | 0.6 |
| RPR*sourceConf | 1.03 | 0.98 | 1.08 | 0.3 |
| Familiarity*sourceConf | 1.05 | 0.99 | 1.10 | 0.09 |
| RPR*task_RateBet | 1.09 | 0.98 | 1.20 | 0.1 |
| Familiarity*task_RateBet | 0.90 | 0.79 | 1.02 | 0.1 |
| sourceConf*task_RateBet | 1.10 | 0.98 | 1.23 | 0.1 |
| RPR*familiarity:sourceConf | 1.01 | 0.96 | 1.07 | 0.6 |

Table S13. LME 2.4 – Online Cohort – Lure items, Betting-Rating Order

| Term | OR | 95% CI LL | 95% CI UL | P-value |
| --- | --- | --- | --- | --- |
| (Intercept) | 0.55 | 0.38 | 0.80 | 0.002 |
| RPR | 1.02 | 0.96 | 1.10 | 0.5 |
| familiarity | 1.08 | 0.97 | 1.21 | 0.2 |
| sourceConf | 1.01 | 0.93 | 1.09 | 0.8 |
| RPR*familiarity | 1.00 | 0.94 | 1.07 | 0.9 |
| RPR*sourceConf | 1.05 | 0.98 | 1.12 | 0.2 |
| familiarity*sourceConf | 1.04 | 0.97 | 1.12 | 0.3 |
| RPR*familiarity:sourceConf | 0.99 | 0.92 | 1.06 | 0.8 |

Table S14. LME 2.4 – Online Cohort – Lure items, Rating-Betting Order

| Term | OR | 95% CI LL | 95% CI UL | P-value |
| --- | --- | --- | --- | --- |
| (Intercept) | 0.65 | 0.38 | 1.11 | 0.1 |
| RPR | 1.12 | 1.04 | 1.20 | 0.003 |
| familiarity | 0.97 | 0.90 | 1.06 | 0.5 |
| sourceConf | 1.11 | 1.02 | 1.20 | 0.01 |
| RPR*familiarity | 1.02 | 0.95 | 1.11 | 0.6 |
| RPR*sourceConf | 1.01 | 0.94 | 1.09 | 0.8 |
| familiarity*sourceConf | 1.05 | 0.97 | 1.13 | 0.2 |
| RPR*familiarity*sourceConf | 1.05 | 0.97 | 1.13 | 0.2 |
