# Supplementary material for: Influences of familiarity and recollection on value-based decision-making
Source: PLoS One. 2025 May 14;20(5):e0322632. doi: 10.1371/journal.pone.0322632 (PMC12077727; doi:10.1371/journal.pone.0322632)
Supplement: Attachment — Submitted filename: response_membet_plos_R1.docx [file pone.0322632.s003.docx]

**Influences of familiarity and recollection on value-based decision-making**

**Response To Reviewers**

We would like to thank the two reviewers and reviewing editor for considering our manuscript and providing detailed and constructive comments. In this revision, we have made several changes in response to these reviews, and we believe these have substantially strengthened our manuscript. Please find our response to the reviewers’ specific comments below. Comments from the reviewers are written in black, bolded and italicized text, while responses from the authors are written in blue text. Text that is quoted from the manuscript is in black with additions written in red.

***Reviewer #1***

***These two experiments (one fully online and the second both online and in-laboratory) examined different memory processes (i.e., familiarity versus recollection) interact during value judgment and risky decision-making. The two experiments were well executed, aimed to answer the 4 research hypotheses and the results obtained were promising, and positive which are relevant for implementation of programs. The statistical analyses were appropriate in testing the various hypotheses postulated.***

Thank you for putting in the time to review our manuscript. We appreciate your feedback and the opportunity to improve on this work.

***1. A pilot study was carried out. Perhaps, a brief summary of its findings would be good to illustrate to the readers about this results and how were these incorporated in the actual experiments.***

We have added more detail describing the pilot study, and the relevant differences between the experiments described here and that original pilot. This information is now described in the methods section for Experiment 1:

The sample size for this experiment was based on in-laboratory pilot data. In that pilot study, participants completed a task very similar to that described here, with the main differences being that there were no lure items, and participants did not provide familiarity ratings, or make explicit judgments about the source of presented items. Instead, we simply focused on the effect of repeated presentations at encoding on subsequent betting rates. In this pilot, we found a simple effect of item repetition during encoding on betting rates, where the expected effect size was adjusted for an expectation of increased variance in an online sample (estimated Cohen’s *d* = 0.44, alpha = 0.05, power = 80%).

***2. In Experiment 1 the analysis showed that there was no main effect of overall source accuracy, nor any significant interaction between source accuracy, RPR and source confidence. What could be the reasons for this? Nor was there any significant interaction between familiarity, RPR, and source confidence.***

Thank you for raising this point. We suspect that the absence of any relationship between betting rates and source accuracy, or significant interactions between source accuracy and RPR and source confidence, is likely a function of the design of experiment 1. In particular, participants only made source judgments in experiment 1 when they judged items to be ‘old’ in the familiarity judgement phase. This feature of the task design likely reduced variance in source accuracy and source confidence judgements, and the number of trials available to statistically test for effects of source accuracy and its interactions with RPR, familiarity and source confidence. We now mention these potential issues in the discussion of experiment 1:

Importantly, however, limitations in the design of this first experiment complicate strong conclusions. In particular, the memory judgments were made immediately after participants made a bet or pass decision. This close temporal proximity could increase the chance that participants’ memory judgments would be influenced by their choice (rather than the opposite), complicating interpretation of any causal role for these memory signals during choice. Second, by thresholding source memory judgments using familiarity, we reduced the amount of data available to test the effects of source memory. This choice may have also limited variance along dimensions like source accuracy and source confidence, possibly explaining why betting rates did not vary with these measures, and why we did not observe an expected increase in source accuracy with repetitions. This choice to threshold source memory judgements by familiarity may have also encouraged a strategy by which weaker items were rated as even less familiar to avoid a cognitively demanding source decision task. This design al­so limited the range of familiarity signals included in these analyses to items that were perceived as ‘old,’ which hampered our ability to test the interaction of familiarity and source memory on decision-making. These limitations similarly impacted our ability to test the role of false source memories for lure items.

***3. Same experiment design was use in Experiment 2. How were these participants split into 2 groups (online and in-laboratory groups)?***

Participants in the online and in-laboratory samples were recruited through separate mechanisms. Participants in the online sample were recruited through Amazon Mechanical Turk (MTurk), while participant in the in-laboratory sample were recruited through local advertisements and contacting participants who had participated in other past studies in our laboratory in the community around Brown University. Thus, these participants were not randomized into online and in-laboratory groups from the same population, but comprised two distinct population samples. To clarify this, we have updated the participants subsection in the methods section for experiment 2 with the following:

Two samples were recruited for this experiment through separate mechanisms: an online sample recruited through MTurk and an in-laboratory sample that was recruited from the local community at Brown University.

***4. For the online sample, majority of the participants were not compliant with the task instructions. Why is this so?***

This is an important point. There are several likely reasons for the poor compliance of the online sample in experiment 2. We discuss these possibilities in the discussion section for that experiment as follows:

As noted above, our online sample did not appear to be as engaged with the memory-based aspects of the task. Unlike Experiment 1, participants had to make source and familiarity judgements for every item, unbroken by other tasks – like the memory-based decisions. This made the memory judgments in Experiment 2 longer and more repetitive and may have contributed to online participants disengaging from the task, reducing the quality of their performance.

The differences between participants’ behavior in the two samples provides an instructive example of the challenges of running online studies with open-ended problems like value-based decision-making, where multiple behavioral strategies might be considered legitimate. For example, participants in the online sample may have considered the marginal value of carrying out recollection to be lower than that of betting randomly, given the time and cognitive effort that a recollection strategy would require – particularly when further time commitment could come at the cost of not completing other online tasks for additional compensation. We further discuss the interpretation of data from the online sample in the supporting material. While it is typical to increase sample size in anticipation of higher inter-individual variance in an online sample, as we did here, this design provides limited protection against the differences in strategies and priorities for online samples compared to those in-laboratory (Stewart et al., 2017).

We also provide a more in-depth discussion of the differences between these samples and the possible reasons for non-compliance in the online sample in the supporting material:

The behavior of this online sample indicated lack of compliance with task instructions. In particular, these participants did not significantly modulate their betting rates according to the RPR. In a value-based decision-making paradigm such as this, there is no one ‘right’ way to complete the task, as the problem is inherently one of personal choice and subjective assessment. However, we would expect that decisions should be modulated by the RPR– if participants were trying to recall the source of each item during the memory-based decision-making phase. These online participants also performed significantly worse in source memory and familiarity judgments compared to the in-laboratory sample. Thus, the on-line participants seemed to be less engaged with the memory-related aspects of the task and/or less willing to use information stored in memory to make choices. As our hypotheses assumed participants engagement with the task and active use of memory during decision-making, we believe these data have limited value for answering our scientific questions. However, these results do provide some useful insight into how small differences in task design and administration can substantially alter results, which we explore below.

There are many potential reasons for these differences from the in-laboratory sample, who completed the same experiment, and the participants in Experiment 1 who completed a similar task online. The task itself was longer than in Experiment 1, and the memory judgments were more repetitive and had a greater separation from the decision task. Our in-laboratory participants were also monitored by an experimenter throughout the task, while online participants completed the experiment without any external supervision. Online participants are also likely more motivated to complete experiments quickly and devote less time and cognitive effort to demanding recollection tasks so that they may complete other tasks and earn more money in a shorter amount of time. The participants in the online sample were also likely more experienced with these kinds of tasks, which may have made them less interested in this particular experiment.

Notably, there were some commonalities in the results for the online and in-laboratory samples. There was a small, albeit noisy, bias towards betting on more subjectively familiar target stimuli in the online sample. The steepness for the relationship between source confidence and familiarity also increased for targets compared to lures, as in the in-laboratory sample. Thus, effects of familiarity might be more easily detectable than those that depend on a more demanding and complicated retrieval process.

The differences between the online and in-laboratory sample indicate important considerations for designing studies examining interactions between memory and decision-making. Future work will need to ensure that participants are equally motivated to engage in the value-based decision-making aspects of the task as well as memory judgments that help support interpretation of decision behavior. These data also speak to how small changes in the way an experiment is conducted can dramatically change the results. Experimenters must exercise caution when interpreting replication failures and consider how seemingly small changes in the sample or task administration can have cascading consequences for compliance with instructions and task strategy.

***5. Discussion and limitations of study were well discussed for both the experiments.***

Thank you. We appreciate your constructive questions and feedback.

***Reviewer #2:***

***This is a well-written and interesting paper examining the separate and interacting effects of recollection and familiarity on value judgment and memory-based decision making. The authors find that familiarity increases subjective value and that it does not tradeoff with source memory—recollection and familiarity instead interact to both increase subjective value. The authors do a great job motivating the study and explaining their hypotheses, and the study design and analyses are well thought through and technically rigorous. The flow of the paper is also great—for example, I had noted two major weakness of experiment one (the causal interpretation of effects of familiarity on choice and the potential unwillingness of participants to complete the source memory task), but both were stated in the discussion of experiment one and then addressed by experiment two. So I think the paper is in generally good shape, and I have the following comments (which are all mostly minor and given in rough order of when they are relevant in the paper) to help improve it:***

Thank you for the useful and encouraging feedback about our manuscript. We appreciate the time and effort that has been put into this review and have found it helpful in improving our manuscript in this revision.

***1. I think it would be more clear to present the prediction figure (Figure 1) after (or alongside) the design of experiment one. This is because the predictions are specific to the experiments, and it would be easier to interpret them once the experiment is explained. I’d like to suggest that the predictions are either added as a separate panel to what is currently Figure 2 (the task design), or that these two figures are reversed in order. I also think that the axes could be improved (the obvious assumption is that left is low and right is high, but this should be made explicit).***

We agree that presenting these predictions later after more information about the experiment has been provided would make it clearer how to interpret this figure. We have taken the suggestion to merge these figures (now Figure 1) and have also added labels to the axes for our predictions.

*2. While reading the task design, I kept wondering how the bet structure made sense when it was introduced by the practice betting task (for example, I kept wondering whether losses were involved). This is later clarified in the memory-based betting task, but it’d be good to introduce that earlier. Related to the presentation of the task, Figure 2C is labeled memory-based betting but in the methods that label is used to describe the whole four-part task. I think this should just be made to match, and the authors can solve this however they see fit/what they think is the most clear. The methods also state the task is three-parts, (which I sort of get if both source tasks are grouped together), but I think it should be labeled as having three-partings.*

**Fig 1. Schematic of task design and predictions. a**. Incidental encoding phase where participants made judgments about whether scenes were indoors (in) or outdoors (out). **b.** Practice betting phase where participants made choices based on explicit risk indicated by a color swatch. **c.** Memory-based betting phase, where risk level had to be inferred from the position of the stimulus during incidental encoding. **d.** Familiarity judgment phase where participants rated the subjective familiarity of each image on a continuous scale from 1-5, **e.** Source judgment phase, where participants selected the previous quadrant in which the image appeared. **f.** Source confidence judgment where participants rated their confidence on a continuous scale from 1-5. Changes in betting frequency predicted by our hypotheses based on **g.** source accuracy, source confidence and retrieved probability of reward (RPR), and **g.** familiarity, source confidence and RPR. **g.** shows positive main effects of source accuracy, source confidence and RPR, and a three-way interaction between all three terms where betting increases when participants are more confident in correctly recalling that the items were associated with a high reward probability source**. h.** shows positive main effects of familiarity, source confidence and RPR, as well as a two-way interaction between familiarity and source confidence, where the impact of familiarity is diminished when participants are more confident in their recollection of the source at encoding.

Thank you for this point. We have modified the description of the practice betting task in the methods to mention the exact amounts at stake for betting and passing:

On each trial, a square swatch in one of these colors was presented in the center of the screen (below the reference grid), and participants were offered the opportunity to either bet on earning a bonus of $5 or earning nothing at the risk-level indicated by the swatch, or choosing to pass and accepting the safe offer of $2

We have also made small changes in wording throughout the manuscript to clarify the distinction between the full experiment inclusive of all the tasks, and the different tasks participants completed (e.g. memory-based betting task, encoding, etc.)

***3. I have a few clarification questions about the statistical analyses for experiment one. How was the categorical lure predictor coded (0 and 1 or some other coding scheme?) Related to this—why was a separate model for the effects of lure familiarity trials needed (LME 1.2), since these effects should be captured by the interaction term of the previous model (i.e. if the categorical lure predictor is 1)? I am not necessarily concerned by this choice, but I would like it to be clarified. There is also a predictor labeled RSP in LME 1.3, but this was not defined as far as I could tell (and I apologize if I missed this).***

We believe this is an important point to clarify. The interaction term in LME 1.1 tests whether the effect of familiarity changes as a function of lure status (e.g. if the relationship between familiarity and betting is weaker, or stronger, for lure items compared to targets). This does not answer the question of whether there is a relationship between familiarity and betting behavior on lure items specifically. That is the question that LME 1.2 is meant to answer. We have clarified these points in the methods section with the following changes:

Generalized linear mixed effects models (LMEs) with a logit link function were used to estimate the contributions of different memory signals to bet or pass decisions in Matlab 2021a (Mathworks, Natick, MA, USA). We first fit a model to data from all trials with the following formula (LME 1.1, in Wilkinson notation):

$$choice \sim1+FM\times lure+\left( 1+FM\times lure \right| participant)$$

Where *FM* corresponds to the familiarity ratings standardized within each participant, *lure* corresponds to a categorical variable coding whether an item was a target or lure (0 or 1). This model also included all main effects for the included terms. This model thus allowed us to test whether betting rates were influenced by familiarity, and if this influence of familiarity depended on whether or not an item was a lure.

To test if betting rates were related to false familiarity on lures specifically, we also modeled the influence of familiarity on choice behavior on lure trials separately using a model with the formula (LME 1.2):

$$choice \sim1+FM+\left( 1+FM \right| participant)$$

Thank you for catching the mistake in LME 1.3. The predictor ‘RSP’ has been changed to ‘RPR.’

***4. It is interesting that repetitions increased the RPR but not source accuracy (and later that the opposite occurs in experiment 2). I think this may be because these variables are in tension with one another: participants should be less accurate about the source if they tend to mis-attribute low probability sources to high probability sources (which should in turn increase the effect of RPR on betting). Was there any evidence for such a relationship? This is also relevant for the overall discussion (~ lines 676 - 682).***

This point highlights an interesting question and interpretation. We believe that the absence of an effect of repetition on source accuracy in experiment 1 likely stems from the task design: namely, that participants only made source judgments on items that they rated as ‘old,’ likely reducing the variability in source memory accuracy among items from both repetition levels (see also our response to point 2 from Reviewer 1). As we mentioned there, we now address this in the discussion of experiment 1:

Importantly, however, limitations in the design of this first experiment complicate strong conclusions. In particular, the memory judgments were made immediately after participants made a bet or pass decision. This close temporal proximity could increase the chance that participants’ memory judgments would be influenced by their choice (rather than the opposite), complicating interpretation of any causal role for these memory signals during choice. Second, by thresholding source memory judgments using familiarity, we reduced the amount of data available to test the effects of source memory. This choice may have also limited variance along dimensions like source accuracy and source confidence, possibily explaining why betting rates did not vary with these measures, and why we did not observe an expected increase in source accuracy with repetitions. This choice to threshold source memory judgements by familiarity may have also encouraged a strategy by which weaker items were rated as even less familiar to avoid a cognitively demanding source decision task. This design al­so limited the range of familiarity signals included in these analyses to items that were perceived as ‘old.’ This hampered our ability to test the interaction of familiarity and source memory on decision-making by reducing the number of trials available for analysis and the range over which these ratings overlapped. These limitations similarly impacted our ability to test the role of false source memories for lure items.

Regarding the potential trade-off in influence between source accuracy and RPR, we believe that the results from the online sample in experiment 2 are inconsistent with this explanation. We observed interaction of RPR and source accuracy in that experiment, indicating that participants were more likely to bet when they were accurate about items coming from a high probability source, and more likely to pass when they were correct about an item coming from a low probability source. This was part of a three-way interaction between source accuracy, RPR and source confidence – where this effect was strongest when participants were more confident about the source of the item. Thus, participants bet on high RPR items less when they made errors about the source compared to when they were correct about the source – even when they were confident in their source judgments.

We believe that the effects of repetition on RPR in experiment 1 likely reflects a confirmatory bias where participants bet more on items that had been repeated and subsequently attributed them to higher value sources. We discuss this possibility in the Discussion section for Experiment 1:

Familiarity and source memory also appeared to become linked through increased value associations for more familiar items. Items with more repetitions were attributed to higher value sources. One possibility for this pattern of behavior is that participants’ source judgments reflect an increase in the value associated with these items in memory which influences post-retrieval evaluative processes. This pattern is similar to that of past studies that have shown that participants attribute greater value to items that they can retrieve from memory (Castel et al., 2012; Rhodes et al., 2017). Another possibility is that these source judgments were confirmatory and reflected the betting behavior of the participants.

We then also discuss this point again in experiment 2, as we observe no effect of repetition on RPR here, and interpret this difference as the result of the confirmatory bias induced by the task design in experiment 1:

Unlike Experiment 1, we did not observe any effect of repetition on RPR, nor the same overall shift toward more frequent association with lower value sources. These results indicate these effects in Experiment 1 were more likely the result of a confirmatory bias to make source judgments consistent with betting choices than a shift toward associating strongly encoded items with higher value sources. These results strengthen the case that the influence of familiarity on expected value exists independent of changes in memory of item-value associations. These findings highlight the importance of separating choice and memory probes to limit the influence confirmatory biases from influencing participants’ judgments.

***5. A nice feature of this design is that it is rational to bet on every trial if participants have no source memory. But isn’t it the case that participants who may be completely rational but not rely on memory are being excluded by the pre-registered criteria (>99% bet rates)? I’m not very concerned by this because very few participants are actually affected here, but I think it might be worth stating why this rationale was chosen given the design of the task. I am also wondering whether you found any relationship between memory performance and overall betting rates. I know you observed that people are generally risk averse here, but I wonder whether it is also the case that less risk averse people are also more forgetful, which would indicate that they are rational when memory is poor? I think this aspect of the design might allow you to gain more insight into participants’ overall strategies when solving the task.***

This is an insightful point. It is true that without source memory betting on every trial would be rational in this task. However, the rationale for rejecting these data was that the lack of variance in behavior made it impossible to use these data to answer our main questions about the role of familiarity and recollection during memory-based choice. Our design also makes it unclear whether these participants are showing rational decision behavior or simply adopting a ‘bet every trial’ heuristic. One solution to this issue would be to present participants with a wider range of values (or catch trials) so that passing or betting is strictly rational without any source memory in some cases. We now touch on this point in the general discussion section:

In both experiments, absent any source memory, the rational course of action is to bet on every trial as the expected value of the risky option is always higher than the safe option. While only a small subset of participants displayed such behavior, they were rejected from data analysis as the lack of variability in their behavior made it impossible to use their data to test questions about the relationship between information in memory and decision behavior. In the future, this task could be modified to include trials with more extreme value independent of source memory so that betting or passing is strictly rational. Such trials would help distinguish between participants who are adopting a heuristic strategy of betting or passing on all trials from those who are making rational economic choices.

We were also interested in the relationship between betting rates and memory performance across individuals as an exploratory question. Unfortunately, the results of these analyses were ambiguous and hard to interpret, and given their exploratory nature, we did not feel they belonged in the main manuscript. However, in this revision we have now included these results in the supplement and describe them briefly in the main text. To summarize, we found moderately strong negative relationships between betting rates and recognition memory performance (measured as d-prime scores) and source accuracy in Experiment 1. However, these effects were not consistently replicated in in-laboratory sample or online sample in Experiment 2. We now describe these results in the supplement with the following:

***Experiments 1 and 2 – individual differences***

In experiment 1 we found negative relationships between recognition memory performance (measured as d-prime scores) and mean overall betting rates (Pearson’s correlation: r(42) = -0.39, P = 0.009), as well as between source accuracy and betting rates (r(42) = -0.50, P = 0.0005). However, we did not find the same relationship for recognition memory in the in-laboratory sample for experiment 2: (d-prime and betting rates: r(34) = 0.15, P = 0.4), and this effect was trending for source accuracy and betting rates (r(34) = -0.29, P = 0.09). The online sample in experiment 2 showed only a trending relationship between recognition memory and betting rates in the same direction as in experiment 1 (d-prime and betting rates: r(86) = -0.19, P = 0.08), and a weaker but significant relationship between source accuracy and betting rates: r(86) = -0.31, P = 0.003).

**Figure S4**. Scatterplots showing relationship between mean betting rate and performance of familiarity task (d-prime) and source accuracy across two experiments with online and in-laboratory samples. Statistics represent Pearson correlation coefficients.

These results are briefly described in the main manuscript after the discussion section for Experiment 2 with a new section:

**Experiments 1 and 2 - Individual differences analysis**

As an exploratory analysis, we tested whether there was any relationship between participants’ overall risk tolerance during memory-based betting (measured as mean betting rate) and memory fidelity. We expected that participants who performed worse on the memory tasks would be more risk averse during the memory-based betting task, as the information they were relying on might be more degraded and unreliable. Instead, we found that memory performance (as measured by recognition memory d-prime scores and source accuracy) were inversely correlated with betting rate in Experiment 1, but this pattern was not consistently replicated in either the online or in-laboratory samples (Figure S4).

These results are hard to interpret as they run counter to our expectations and are not clearly consistent across experiments in either direction or significance. Furthermore, the fact that this relationship is strongest in the online experiments, particularly Experiment 1 where betting and memory judgements are more likely to be confirmatory, makes us doubt that this relationship is driven by a meaningful connection between memory processes and decision strategy. However, the reviewer’s suggestion that participants with worse memory might be instead following a more rational strategy of betting more often is certainly a plausible alternative explanation. We now briefly describe our interpretation and these alternative possible explanations in the general discussion section:

We also conducted an exploratory analysis examining the relationship between betting rates and memory performance. While we expected that participants with worse memory would bet less frequently, instead we observed the opposite relationship, though this effect was not consistent across experiments and samples. This relationship could arise for several reasons: because these participants were less engaged with the memory components of the task and instead tended toward the more rational strategy of betting more often, or participants who performed worse on these tasks (and perhaps are generally less engaged with the experiment) could also be less risk aversion in general. The fact that these effects are strongest in the online sample in Experiment 1 where the betting and memory component of the task are immediately adjacent also make us suspicious that the correlation between these measures is driven by individual differences in task engagement more than a deeper relationship between memory and these decision processes.

***6. While the authors admit that drawing causal influences of familiarity on betting behavior is difficult, there are still statements about directionality in the results (lines 275-277; lines 281-282). I think these should be amended. Relatedly, in experiment two the authors state that adding some temporal distance (via separate blocks for decisions and memory judgments) can help address this, but I don’t see how this is necessarily the case. The element of the experiment two design that really accomplishes this is the counterbalancing, so in my opinion the writing should be revised to reflect that as a primary explanation for this design choice in experiment two.***

We agree that inferring causal influence of familiarity on betting rates is challenging, particularly in Experiment 1. We have revised the language in the paragraph pointed out by the reviewer to the following:

Familiarity could influence value judgments in this task in at least two ways: it could differentiate target items from lures, and thus provide a clue that an item is associated with some source and hence have some probability of being rewarded. Familiarity could also confer increased value to an item through direct integration into an expected value signal. As predicted by our first hypothesis, there was a strong relationship between betting rates and familiarity (Figure 3a), with participants tending to bet more on items that they had rated as highly familiar (OR = 2.93, 95% CI: 2.04-4.19, P < 0.001). Participants also bet less on lure items compared to targets overall (OR = 0.78, 95% CI: 0.66-0.92, P = 0.003). Nonetheless, the relationship between betting rates and familiarity was maintained for lure items when tested on their own (OR = 1.50, 95% CI: 1.08-2.08, P = 0.01), and there was no interaction between target/lure status and familiarity. (OR = 0.81, 95% CI: 0.57-1.13, P = 0.2). Thus, participants’ betting rates increased along with perceived familiarity, even in absence of exposure (Figure 3b).

We have also made changes throughout the manuscript to modify where the language could be interpreted as suggesting a causal inference where this is not strictly possible.

To the second point regarding the relative importance of counterbalancing and temporal distance between memory judgements and the betting phase – we believe that both of these factors are probably important for controlling the circular dependencies between participants’ choices and their ratings. However, neither methodological change can fully prevent such circular influence. Counterbalancing does not fully protect against this problem as participants’ ratings could reflect a confirmation of their betting behavior, or their betting could reflect a confirmation of their ratings. Similarly, the temporal distance between the rating and choice might help reduce a confirmatory bias but cannot eliminate the possibility (as participants could always recall what they did in a previous part of the experiment). We have updated some the language describing the rationale for Experiment 2 to reflect this:

In Experiment 2, we aimed to replicate and extend the results of the first experiment by making changes to the task that would address the limitations described above. First, memory judgments and betting decisions were presented in two separate phases, reducing the potential for participants to make memory judgments that conform to their decisions. Second, the order of memory judgments and betting decisions were counterbalanced across participants, so that we might control for any order effects for these two tasks and potential confirmatory biases for making choices consistent with rating and vice-versa. Third, participants made familiarity and source memory judgments in two separate phases of the experiment, and queries about the source were made about all items, old and new, regardless of their rated familiarity. This allowed us to break the interdependency between familiarity ratings and source judgments.

***7.Regarding the statistical methods for experiment two, I am again unclear why a separate model for lure items is needed, since this should again (I think) be captured by the interactions with the lure categorical predictor variable. I understand why this is the case for targets since the authors need to look at the effects of source accuracy, but the rationale for doing so with lures should be made more clear.***

Our response to point 3 also applies here. This LME model was necessary to test the relationship of familiarity ratings and choice behavior on lure items specifically. We have clarified this in the methods section as follows:

We tested a similar model for lure items (LME 2.3), though without the source accuracy factor. As in the previous experiment, this model allowed us to test for relationships between these different factors and betting behavior specifically on lure trials alone.

***8. When comparing the linear and exponential models, a procedure that is more robust to overfitting should be used. Ideally this would be cross validation (for example, leave-N-subjects or, per subject, leave-N-trials out). I think it is possible that this may reveal more nuanced differences in the fit than just a straight comparison of the NLL. This ultimately may not be so important given that the authors state (but don’t show) that a similar result was observed for the relationship between familiarity/source confidence and lures/errors/correct responses, but I think cross validation would strengthen this result.***

We agree that cross-validation methods might provide a more robust comparison of the exponential and linear functions. We have repeated this analysis using a 2-fold cross-validation procedure, as suggested. This analysis yielded the same pattern of results observed when comparing these data using the negative log-likelihood for target items with correct source judgements. We have updated the results section to reflect this change:

We first assessed whether the relationship between source memory and familiarity could be better explained by an exponential or linear function. We focused this analysis on correct source judgments of target items, as we anticipated that the recollective process would be most pronounced within a subset of these trials. Using a 2-fold cross-validation procedure, we fit linear or exponential functions to these data and tested them in the other half of the data. Neither model was strongly preferred based on the total negative log-likelihood across participants averaged across fold permutations, though if anything, the linear model provided a slightly better fit (linear = 1275.8, exponential = 1275.9). Comparison of the negative log-likelihood of each model for individual participants also indicated that the linear model was better fit for only a slightly larger percentage of participants (66.7%). The relationship between familiarity and source confidence was thus not obviously better fit by either function, though the linear function was slightly preferred.

Please also see changes to the methods section reflecting this update:

We also conducted secondary analyses examining the relationship of familiarity and source confidence. Exponential and linear functions were fit to the relationships between familiarity and source confidence ratings. In both cases, the functions were fit using the Matlab function ‘fminunc’ with a cost function based on the negative log likelihood estimated using a Gaussian probability density function. To compare the linear and exponential functions, we carried out a 2-fold cross-validation procedure where we randomly assigned half of the trials to either training and test sets, fit the function in the training set and then estimated the negative log-likelihood for the estimated parameters in the test set. We repeated this procedure 100 times for each participant so that different combinations of trials could be used in either training to test sets. To avoid local minima, the models were fit 10 times for each participant with the parameters initialized at starting points randomly sampled from independent standard normal distributions.

We also used the same cross-validation procedure to test if an exponential or linear function fit this relationship better for target items with source errors (a better fit for the linear function in 58% of participants), though the result was more ambiguous for lure items (linear was preferred for 50% of participants). Thus, these results were largely the same results as we found when comparing these functions using the negative log-likelihood.

***- A few typos:
- Stars are missing from the source accuracy portion of table 2
- line 235: they value expectations —> their value expectations
- line 525: I think this should be referencing Figure 4a rather than 3a (which was for experiment 1)
- line 528: Same here, I think this should be Figure 4b instead of 3b
- line 547: Figure 3c —> Figure 4c
- line 559: Figure 3d —> Figure 4d***

Thank you for these detailed notes. We have made changes where typos and mistakes were identified.
